# Impact of oral/dental disease burden on postoperative infective complications: a prospective cohort study

**DOI:** 10.1007/s00784-023-05251-4

**Published:** 2023-09-20

**Authors:** Hanako Suenaga, Mark Schifter, Nancy Chen, Farheen Ali, Karen Byth, Chris Peck

**Affiliations:** 1 Sydney Dental School, Faculty of Medicine and Health, The University of Sydney, Level 2-3 Westmead Centre for Oral Health, Westmead, NSW 2145 Australia; 2 Division of Advanced Prosthetic Dentistry, Tohoku University Graduate School of Dentistry, 4-1 Seiryo-Machi, Aoba-Ku, Sendai, 980-8575 Japan; 3 Department of Oral Medicine, Oral Pathology and Special Needs Dentistry, Westmead Centre for Oral Health, Level 3, Westmead, NSW 2145 Australia; 4 Department of Anaesthesia and Perioperative Medicine, Westmead Hospital, Cnr Darcy & Hawkesbury Roads, Westmead, NSW 2145 Australia; 5 Research and Education Network, Western Sydney Local Health District, Westmead Hospital, Westmead, NSW 2145 Australia

**Keywords:** Postoperative infective complications, Preoperative dental care, Oral bacteria

## Abstract

**Objectives:**

This prospective cohort study aimed to assess the association between dental disease burden and postoperative infective complications (POICs) in patients undergoing major surgical procedures under general anaesthesia.

**Methods:**

Pre-surgical dental assessment was undertaken on patients planned for major surgery. Demographic and surgical variables including putative risk factors for POICs and POIC status were documented. The univariable association between POIC status and each factor was examined. Those variables associated at *P* value ≤ 0.2 were candidates for inclusion in multiple logistic regression models. Backward stepwise variable selection was used to identify the independent predictors for POIC in the best fitting logistic regression model. The area under the receiver operating curve (AUC) was used to quantify the model’s global classification performance.

**Results:**

Among the 285 patients, 49 patients (17.2%) had POICs. The independent predictors for POIC were expected length of hospital stay (4–6 days; odds ratio [OR] = 4.80, 95% confidence internal [CI]: 1.30–17.70, *P* = 0.018, 7–9 days; OR = 5.42, 95% CI: 1.51–19.41, *P* = 0.009, ≥ 10 days; OR = 28.80, 95% CI: 4.12–201.18, *P* < 0.001), four or more decayed teeth (OR = 6.03, 95% CI: 2.28–15.94, *P* < 0.001) and visible tongue plaque (OR = 3.21, 95% CI: 1.54–6.70, *P* = 0.002). The AUC was 0.78 (95% CI: 0.71–0.85) indicating good discrimination. A simple screening tool for POIC was developed.

**Conclusions/Clinical relevance:**

In addition to systemic/surgical factors, this study identified clinically detected decayed teeth and visible tongue plaque as independent predictors for POICs. Preoperative dental assessment/care might be beneficial to assess risk for POICs and improve postoperative outcomes.

**Supplementary Information:**

The online version contains supplementary material available at 10.1007/s00784-023-05251-4.

## Introduction

The links between oral and general health have been increasingly recognised over the past two decades [1, 2]. Recognition of this relationship and, in turn, addressing the burden of dental disease, may serve to prevent disease and disability and reduce health care costs. Several studies have indicated that providing dental care before major surgical procedures facilitated by means of general anaesthesia, may prevent postoperative infective complications (POICs) including postoperative pneumonia and surgical site infection [3–11]. Recent reviews and studies suggest that perioperative chlorhexidine mouthwashes may significantly decrease the incidence of postoperative pneumonia in patients undergoing elective cardiac surgery [4–6] and non-cardiac surgery [7]. It has also been reported that preoperative dental care provided by oral health practitioners (i.e. oral health therapists, dental hygienists, dentists) had a significant positive effect in reducing adverse postoperative outcomes, including lessening the incidence or risk for POICs [8–11].

Following major surgery, infectious complications are one of the main causes of postoperative morbidity and mortality, with consequent extended post-operative length of stay beyond the planned discharge date contributing to increased financial costs to the healthcare system [12, 13]. It follows then, that it is crucial to reliably predict postoperative complications in order to prevent infectious complications and to improve overall patient care [14]. For this reason, a variety of systemic or operative risk factors for postoperative complications have been identified in various reports including clinical guidelines such as the Global Guidelines for the Prevention of Surgical Site Infection [15]. Disappointingly, findings about the impact of oral/dental disease factors including the burden of dental disease adversely affecting postoperative outcomes are limited, despite multiple studies reporting a clear positive impact of pre-operative oral care on postoperative outcomes [3–11]. Several studies have reported that presence and severity of dental infection and inflammation [16–18] could be predisposing factors for postoperative pneumonia and surgical site infection [19]. However, as these studies were narrowly focused on only a single oral/dental disease factor or surgical outcome and as most of these subject population**s** had also received pre-operative oral/dental care, clear associations between the level of oral/dental health and POICs have not been fully evaluated. Therefore, the aim of this study was to assess the association between dental disease burden and POICs in patients undergoing major surgical procedures under general anaesthesia. The null hypothesis was that there was no difference in POICs in patients with poor oral health status when compared to those with good oral health.

## Methods

### Study design

We performed a prospective cohort study of patients, at Westmead Hospital, Australia, from December 2018 to March 2021, who underwent major surgical procedures under general anaesthesia. The institutional ethics review board of the hospital approved this study (No. LNR/17/WMEAD/579). Patients were included if they were at least 20 years old, underwent surgery under general anaesthesia and required overnight postoperative hospitalisation. Surgical patients were approached sequentially until the target of 300 consented and had dental assessments performed at the time of their pre-anaesthetic consult through the Department of Anaesthetics Pre-Admissions Clinic, Westmead Hospital. Patient hospital records were reviewed to identify patient characteristics and their postoperative outcomes.

### Preoperative dental assessment

Dental assessment consisted of extra-oral and intra-oral examination. To determine the level of oral disease, we used the following standardised indices: (a) Decayed, Missing and Filled Teeth Index (DMFT) [20], (b) Periodontal Screening and Recording Index (PSR) [21], (c) Oral Hygiene Index [22], (d) Tongue Plaque Index [23] and (e) The Challacombe Scale which serves as the Clinical Oral Dryness Score [24], as described in Appendix Table 1. Dental examination was performed by three dentists who were trained and calibrated accordingly for evaluating the above indices including detecting a carious lesion using the WHO criteria [20]. The examiners recorded a tooth as decayed only if it presented with detectably softened floor, undermined enamel or a softened wall. According to these criteria, all the stages that precede cavitation as well as other conditions similar to the early stages of a carious lesion were considered sound [20]. Oral hygiene was assessed by visible tongue plaque (coating) for all patients including fully edentulous cases accepting this may also relate to the degree of patient salivary hypofunction (which we termed “oral dryness”).

### Study outcome

The outcome of this study was POIC, viz. surgical site infection, sepsis, postoperative pneumonia, methicillin-resistant Staphylococcus aureus infection, urinary tract infection, and infective endocarditis, as diagnosed and managed by the surgical team.

### Patients’ characteristics

Based on previous literature [12, 25, 26], the following demographic characteristics were documented from review of the medical records to determine their influence on POICs; gender, age, education, body mass index, smoking status (non-smoking, past-smoking, or present-smoking), glycated hemoglobin and physical status including the presence of comorbidities as assessed by the American Society of Anesthesiologists (ASA) physical status classification system [27]. The surgical factors assessed were expected length of stay after surgery measured in days. Comorbidity variables identified included cardiovascular and/or respiratory diseases as described in the International Classification of Diseases, 11th Revision [28].

### Statistical analysis

IBM SPSS Statistics version 28 (IBM, Armonk, NY, USA) was used to analyse the data. Continuous variables were summarized using the median and interquartile range (lower quartile, upper quartile). Frequencies and percentages were used for categorical variables. Chi-squared or exact permutation tests as appropriate were used to test for association between each categorical variable and the dichotomous outcome of interest, namely POIC status (present versus absent). Mann–Whitney tests were used for each continuous variable.

Those variables demonstrating univariable association (*P* < 0.2) with POIC status were candidates for inclusion in multiple logistic regression (LR) models. Backward stepwise variable selection was used to identify the independent predictors of POIC status in the best fitting multiple logistic regression model (MLR). Adjusted odds ratios (OR) with 95% confidence intervals (CIs) were used to quantify the strength of association with POIC. Boxplots were used to illustrate the distribution of the probability of infection predicted using the best fitting model by POIC status.

A simple risk score for POIC in the study population was created by rounding the regression coefficients to the nearest integer in the best fitting MLR model. The area under the receiver operating curve (AUC) was used to quantify the global performance of this score and that of the linear predictor from the best MLR model to correctly classify a patient’s POIC status. In this observational study all analyses were exploratory and 2-tailed tests with a significance level of 5% were used throughout.

## Results

**Fig. 1:**
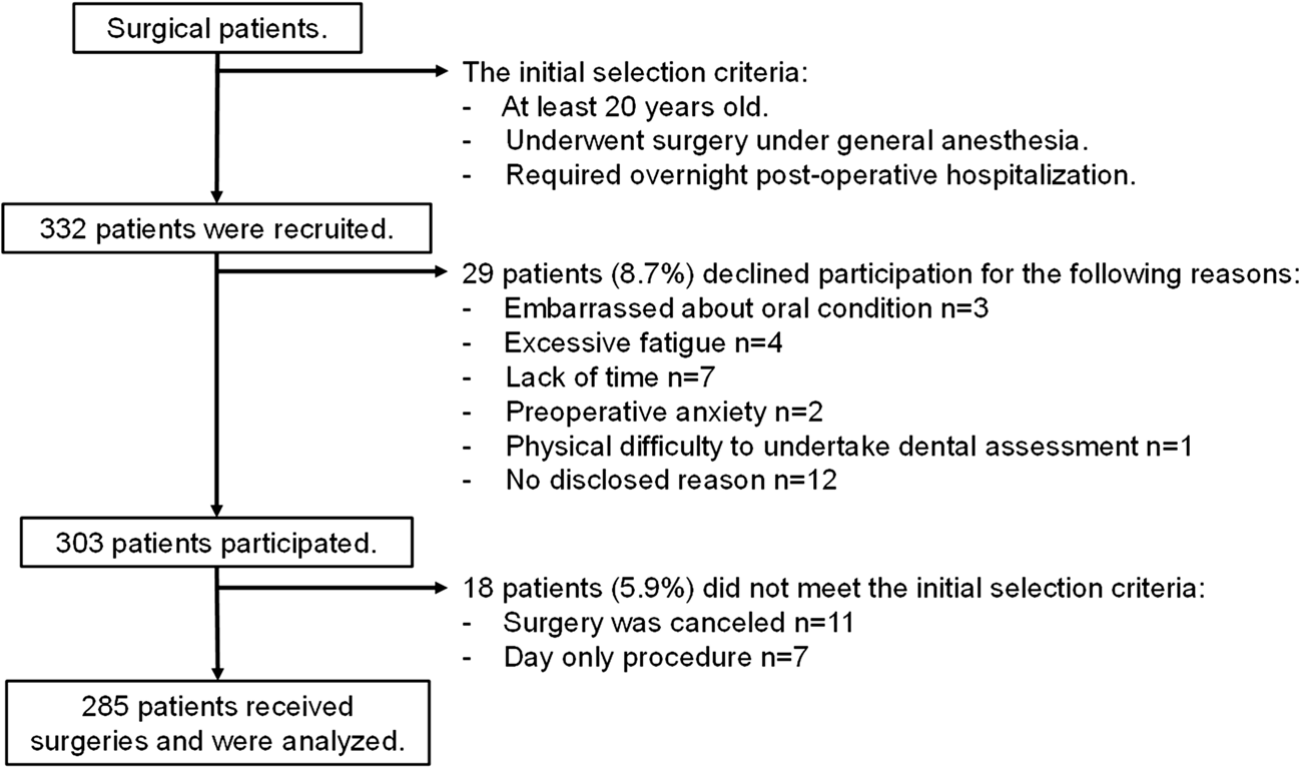
Subject selection decision tree. ASA, the American Society of Anesthesiologists physical status classification

From November 2018 to February 2021, 332 subjects who were screened for the inclusion criteria were recruited and 303 (91.3%) of those agreed to participate in the study and underwent a dental assessment (Fig. 1). Of these patients, 18 patients did not meet the inclusion criteria (e.g. due to the cancelation of surgery) and the remaining 285 patients were evaluated.

Tables 1 and 2 show distribution of categorical and continuous variables by POIC status. Whilst 49 out of 285 (17.2%) patients acquired POICs in total, a significantly higher rate of POICs was observed among patients with longer expected hospital stays, decayed teeth, higher PSR, visible tongue plaque, and dry mouth.

**Table 1:** Distribution of categorical variables by postoperative infective complication status

Variable	Values taken	Total	Postoperative infective complication				*P*-value*
			No		Yes		
		*N*	*n*	%	*n*	%	
Age	< 60	127	109	85.8%	18	14.2%	0.226
	≥ 60	158	127	80.4%	31	19.6%	
Gender	Male	130	104	80.0%	26	20.0%	0.250
	Female	155	132	85.2%	23	14.8%	
Education	Primary	26	24	92.3%	2	7.7%	0.916
	Secondary	203	166	81.8%	37	18.2%	
	Tertiary	51	42	82.4%	9	17.6%	
ASA	< 3	127	110	86.6%	17	13.4%	0.127
	≥ 3	158	126	79.7%	32	20.3%	
Expected hospital stay (days)	1	49	45	91.8%	4	8.2%	0.009
	2–3	106	93	87.7%	13	12.3%	
	4–6	61	49	80.3%	12	19.7%	
	7–9	60	44	73.3%	16	26.7%	
	≥ 10	9	5	55.6%	4	44.4%	
BMI	18.5–24.9	65	57	87.7%	8	12.3%	0.493
	25–29.9	96	78	81.3%	18	18.8%	
	≥ 30	124	101	81.5%	23	18.5%	
Smoking status	Non	153	127	83.0%	26	17.0%	0.905
	Past	88	74	84.1%	14	15.9%	
	Current	42	34	81.0%	8	19.0%	
HbA1c	< 7	105	81	77.1%	24	22.9%	0.662
	≥ 7	26	19	73.1%	7	26.9%	
Circulatory disease comorbidity	No	108	92	85.2%	16	14.8%	0.406
	Yes	177	144	81.4%	33	18.6%	
Respiratory disease comorbidity	No	231	193	83.5%	38	16.5%	0.492
	Yes	54	43	79.6%	11	20.4%	
Number of decayed teeth	0	168	147	87.5%	21	12.5%	< 0.001
	1	39	36	92.3%	3	7.7%	
	2–3	42	32	76.2%	10	23.8%	
	≥ 4	27	15	55.6%	12	44.4%	
Number of missing teeth	< 8	173	144	83.2%	29	16.8%	0.956
	≥ 8	103	86	83.5%	17	16.5%	
Number of filled teeth	< 8	188	156	83.0%	32	17.0%	0.754
	≥ 8	53	43	81.1%	10	18.9%	
PSR average	< 2	118	105	89.0%	13	11.0%	0.013
	≥ 2	121	93	76.9%	28	23.1%	
Oral Hygiene Index	< 2.6	92	79	85.9%	13	14.1%	0.327
	≥ 2.6	147	119	81.0%	28	19.0%	
Tongue Plaque Index	Non Visible	161	143	88.8%	18	11.2%	0.003
	Visible	117	88	75.2%	29	24.8%	
Dry mouth	No	169	149	88.2%	20	11.8%	0.005
	Yes	109	82	75.2%	27	24.8%	
Edentulous	No	240	199	82.9%	41	17.1%	0.632
	Yes	36	31	86.1%	5	13.9%	

*ASA* the American Society of Anesthesiologists physical status classification, *BMI* body mass index, *PSR* Periodontal Screening and Recording Index

*Chi-squared or exact permutation test

**Table 2:** Distribution of continuous variables by postoperative infective complication status

Variable	No postoperative infective complication			Postoperative infective complication			Mann–Whitney *P*-value
	Median	Percentile 25	Percentile 75	Median	Percentile 25	Percentile 75	
Age	61.0	47.5	71.0	64.0	49.0	75.0	0.154
Pre-surgical ASA	3.0	2.0	3.0	3.0	2.0	3.0	0.164
Expected length of stay	3.0	2.0	5.0	5.0	3.0	7.0	0.001
Pre-surgical BMI	28.7	25.2	35.3	29.2	26.5	34.9	0.723
Hba1c	5.9	5.3	6.9	6.0	5.5	6.8	0.581
Number of decayed teeth	0.0	0.0	1.0	1.0	0.0	4.0	0.001
Number of missing teeth	6.0	1.0	17.0	5.5	1.0	16.0	0.908
Number of filled teeth	2.0	0.0	7.0	3.0	0.0	8.0	0.505
PSR average	1.8	1.3	2.0	2.0	1.7	2.3	0.019
Oral Hygiene Index	3.0	1.7	4.8	3.7	2.5	4.7	0.222
The cumulative score	0.0	0.0	1.0	1.0	0.0	2.0	0.003

*ASA* the American Society of Anesthesiologists physical status classification, *BMI* body mass index, *PSR* Periodontal Screening and Recording Index

Eleven candidate variables demonstrating univariable association (*P* < 0.2) with POIC status (pre-surgical ASA, ASA ≥ 3, pre-surgical BMI, PSR average, PSR ave ≥ 2, the cumulative score, dry mouth, Tongue Plaque Index, edentulous, expected length of stay categorised into 5 groups, number of decayed teeth categorised into 4 groups) were input into multiple logistic regression analysis (Table 3). The independent predictors for POIC were expected length of hospital stay (4–6 days; odds ratio [OR] = 4.80, 95% confidence internal [CI]: 1.30–17.70, *P* = 0.018, 7–9 days; OR = 5.42, CI: 1.51–19.41, *P* = 0.009, ≥ 10 days; OR = 28.80, CI: 4.12–201.18, *P* < 0.001), four or more decayed teeth (OR = 6.03, 95% CI: 2.28–15.94, *P* < 0.001) and visible tongue plaque (OR = 3.21, 95% CI: 1.54–6.70, *P* = 0.002).

**Table 3:** Unadjusted and adjusted* odds ratios with 95% CIs for postoperative infective complications

Variable	Values taken	Unadjusted odds ratio	95% CI for OR		*P*-value	Adjusted* odds ratio	95% CI for adj OR		*P*-value
			Lower	Upper			Lower	Upper	
Age	< 60	1	Reference category						
	≥ 60	1.48	0.78	2.79	0.228				
Gender	Male	1	Reference category						
	Female	0.70	0.38	1.29	0.252				
Education	Primary	1	Reference category						
	Secondary	2.68	0.61	11.82	0.194				
	Tertiary	2.57	0.51	12.89	0.251				
ASA	< 3	1	Reference category						
	≥ 3	1.64	0.87	3.12	0.129				
Surgical duration	< 120 min	1	Reference category						
	≥ 120 min	1.88	0.95	3.73	0.071				
Expected hospital stay (days)	1	1	Reference category			1	Reference category		
	2–3	1.57	0.49	5.10	0.45	2.12	0.59	7.60	0.251
	4–6	2.76	0.83	9.16	0.098	4.80	1.30	17.70	0.018
	7–9	4.09	1.27	13.21	0.018	5.42	1.51	19.41	0.009
	≥ 10	9.00	1.70	47.60	0.01	28.80	4.12	201.18	< 0.001
BMI	18.5—24.9	1	Reference category						
	25—29.9	1.64	0.67	4.05	0.279				
	≥ 30	1.62	0.68	3.86	0.274				
Smoking status	Non	1	Reference category						
	Past	0.92	0.45	1.88	0.828				
	Current	1.15	0.48	2.77	0.756				
HbA1c	< 7	1	Reference category						
	≥ 7	1.24	0.47	3.31	0.663				
Circulatory disease comorbidity	No	1	Reference category						
	Yes	1.32	0.69	2.53	0.407				
Respiratory disease comorbidity	No	1	Reference category						
	Yes	1.30	0.62	2.75	0.493				
Number of decayed teeth	0	1	Reference category			1	Reference category		
	1	0.58	0.17	2.06	0.403	0.46	0.12	1.75	0.257
	2–3	2.19	0.94	5.09	0.069	2.36	0.95	5.88	0.064
	≥ 4	5.60	2.31	13.58	< 0.01	6.03	2.28	15.94	< 0.001
Number of missing teeth	< 8	1	Reference category						
	≥ 8	0.98	0.51	1.89	0.956				
Number of filled teeth	< 8	1	Reference category						
	≥ 8	1.13	0.52	2.49	0.754				
PSR average	< 2	1	Reference category						
	≥ 2	2.43	1.19	4.97	0.015				
Oral Hygiene Index	< 2.6	1	Reference category						
	≥ 2.6	1.43	0.70	2.93	0.328				
Tongue Plaque Index	Non Visible	1	Reference category			1	Reference category		
	Visible	2.62	1.37	4.99	0.030	3.21	1.54	6.70	0.002
Dry mouth	No	1	Reference category						
	Yes	2.45	1.30	4.64	0.006				
Edentulous	No	1	Reference category						
	Yes	0.78	0.29	2.13	0.632				
Age per 10 year increase		1.14	0.93	1.39	0.210				
Pre-surgical ASA per unit increase		1.33	0.87	2.04	0.182				
Expected length of stay per day increase		1.21	1.08	1.34	< 0.001				
Pre-surgical BMI per 5 unit increase		0.98	0.79	1.21	0.830				
HbA1c per 0.5 increase		1.09	0.91	1.29	0.325				
Number of decayed teeth per unit increase		1.19	1.05	1.35	0.008				
Number of missing teeth per unit increase		0.99	0.97	1.03	0.893				
Number of filled teeth per unit increase		1.03	0.97	1.09	0.287				
PSR average per unit increase		1.41	0.97	2.05	0.072				
Oral Hygiene Index per unit increase		1.10	0.96	1.25	0.177				
The cumulative score		1.25	1.05	1.50	0.015				

*Candidate variables for inclusion were pre-surgical ASA, ASA ≥ 3, pre-surgical BMI, PSR average, PSR average ≥ 2, the cumulative score, dry mouth, Tongue Plaque Index, edentulous, expected length of stay grouped, number of decayed grouped

**Fig. 2:**
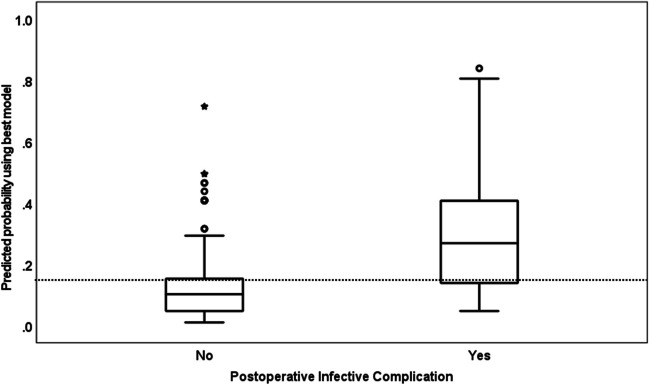
Boxplots of the predicted probability of postoperative infective complication

Predicted probabilities from the best fitting model were illustrated in boxplots (Fig. 2). There is a clear distinction between the group with POIC and that without. By rounding the *β* coefficients for each independent predictor in the best-fitting model, the simple risk score with a range 0–6 in Table 4 was produced.

**Table 4:** The simple risk score for postoperative infective complications

Score		0
	If expected length of stay 2 and 3 days	+ 1
	If expected length of stay 4–9 days	+ 2
	If expected length of stay ≥ 10 days	+ 3
	If number decayed teeth 2 and 3	+ 1
	If number decayed teeth ≥ 4	+ 2
	If visible tongue plaque	+ 1

**Fig. 3:**
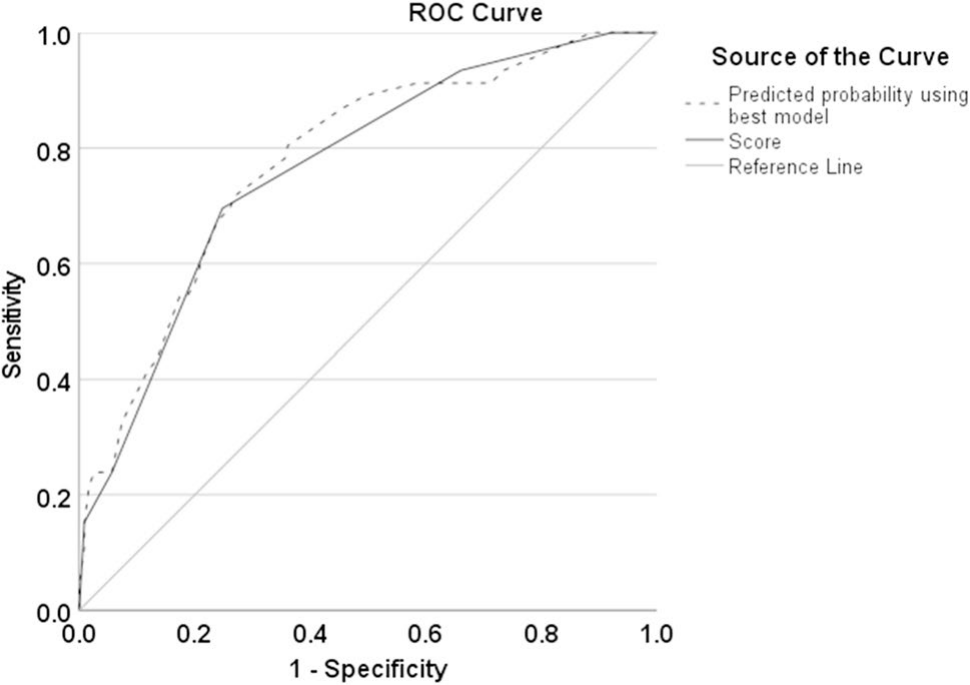
The receiver operated curve (ROC) for the best fitting multiple logistic regression model and the scoring model showing the area under the curve (AUC): 0.78 (95% CI = 0.71–0.85) and 0.77 (95% CI = 0.69–0.84)

Figure 3 shows the receiver operating curves for the linear predictor from the best model and that for the simple score. The associated AUCs were virtually identical, being 0.78 (95% CI 0.71–0.85) and 0.77 (95% CI 0.69–0.84), respectively.

Table 5 shows the performance of the scoring model for this study cohort. The percentage of patients with POICs tended to increase with increasing the score. No patients with a score of 0 had POICs, whilst nearly 80% of those with a score of 5 did.

**Table 5:** Performance of the simple risk score for postoperative infective complications in the patient cohort

Score	Postoperative infective complication		Total number of patients	Percentage of patients with complication
	No	Yes		
	*n*	*n*	*N*	
0	18	0	18	0.0%
1	60	3	63	4.8%
2	95	11	106	10.4%
3	44	21	65	32.3%
4	11	4	15	26.7%
5	2	7	9	77.8%

## Discussion

Whilst previous studies have demonstrated a positive effect of preoperative dental care on preventing POICs, the recommendation of preoperative dental care remains controversial [29, 30] and an optimal protocol/guideline to provide preoperative dental assessment/care has not been developed. As well, the extent of any pre-operative dental intervention remains uncertain. This is because the association between oral health and postoperative outcomes are yet to be fully studied and appreciated. In this prospective cohort study, we identified the number of decayed teeth ≥ 4 and visible tongue plaque as independent predictors for POICs. This finding suggests that preoperative dental assessment may be useful to identify patients at increased risk of postoperative complications and allow perioperative management strategies that improve patient outcomes. The simple risk score created in this study allows health practitioners to simply assess risk for POICs in clinical practice.

Mirzashahi and co-workers/colleagues revealed significant associations between surgical site infection and caries, gingivitis/periodontitis and the presence of active dental abscesses [19], and Bergan and co-workers/colleagues found significant relationships between postoperative pneumonia and tongue plaque and poor denture hygiene [31]. As the study outcome in this study, POICs, included surgical site infection and postoperative pneumonia, our primary finding about the significant association between POICs and the presence of decayed teeth and tongue plaque is in keeping with these previous findings.

There are several possible mechanisms by which the presence of multiple decayed teeth and visible tongue coating could be associated with increased postoperative infections. Firstly, there is mounting evidence that oral bacteria can contribute to POICs [31–33] such as postoperative pneumonia or surgical site infection. Recent reviews suggest that one of the primary causes of postoperative pneumonia is the aspiration of oral and pharyngeal secretions during placement and removal of the endotracheal tube before and after surgery [4, 34]. Akutsu and co-workers identified the same pathogenic bacteria in the postoperative sputum of patients with postoperative pneumonia following esophagectomy as the bacteria isolated from the same patient’s preoperative dental plaque [32]. Also, Nishikawa and colleagues detected the same bacterial strains from both the drainage fluid from the abdominal cavity of patients with peritonitis after gastrectomy and from their periodontal pockets [35]. Furthermore, surgical site infection pathogenesis may be explained by the “Trojan horse mechanism”, which posits that pathogens remote from the surgical site infection area, such as, within or on the teeth, gums, or gastrointestinal tract, can be taken up by immune cells (macrophages or neutrophils) and these “first responders” then travel carrying the ingested bacteria to the wound site where they cause infection [25]. A second possible mechanism could be that the presence of decayed teeth and/or visible tongue plaque serves as a biomarker of poor systemic health and/or indicator of poorer socioeconomic status. Poor oral health is a major contributor to general health conditions, and noting that it has particular associations with cardiovascular disease, diabetes mellitus, cancers, pneumonia, and premature birth [36]. The burden of poor oral health reflects significant social inequalities, between and within countries, disproportionally affecting lower and middle-income countries, and mostly affecting people from lower socioeconomic backgrounds [37]. Other risk groups for poor oral health are those who cannot maintain their oral hygiene on their own due to their age or disability, or who have lower health literacy with regards to both their general and their oral health [37]. Oral diseases share many risk factors with chronic noncommunicable diseases, such as tobacco use, harmful use of alcohol, a high dietary intake of free sugars and poor hygiene [37]. Therefore, decayed teeth and visible tongue coating might serve as a reliable indicator of a wide range of demographic risk factors for POICs. Besides the number of decayed teeth and visible tongue plaque, the multiple regression model identified the length of expected postoperative hospital stay as an independent predictor. This may be because expected postoperative hospital stay could be determined whilst considering multiple patient and surgical factors comprehensively.

One of the key strengths of this study is the simple score which allows health practitioners assess the risk for POICs with just counting the number of decayed teeth and checking visible tongue coating and the length of expected hospital stay. Decayed teeth and tongue plaque for this score can be assessed without any special equipment including radiographs. In this study cohort, an extremely high percentage of patients with score 5 acquired POICs. Since score 5 can be reached only when decayed teeth and/or visible tongue plaque exist, dental assessment prior to surgery would be required to screen those high-risk patients. As there has been significant growth in the demand for surgical services [38, 39], this simple score to assess risk for POICs would be valuable.

There are some limitations with this study. Firstly, the sample size was relatively small compared with the number of patients who underwent a general anaesthetic and a major operation. Secondly, this study focuses on early POICs that occurred within 1 month after surgery, without longer follow-up. Consequently, POICs that may manifest at a later date were not recorded, and the effect of oral disease burden on delayed complications related to oral bacteria such as late-onset infective endocarditis [2] or chronic prosthetic joint infection [40] was not assessed. Thirdly, whilst the simple score was effective in this study population, validation is required in a future study. Finally, although this study revealed the significant association between oral variables and POICs, it did not reveal whether oral variables cause or directly impact on POICs. Future studies will focus on investigating the relationships between preoperative oral microbiome and POICs and effect of preoperative dental care on preventing POICs through a randomized controlled clinical trial.

In conclusion, this study demonstrated the significant associations between oral/dental disease and POICs. Clinically detected decayed teeth and visible tongue plaque were identified as independent predictors for POICs. Preoperative dental assessment/care might be beneficial to improve postoperative outcomes.

### Supplementary Information

Below is the link to the electronic supplementary material.

Supplementary file1 (DOCX 53 KB)

## Data Availability

The data that support the findings of this study are available from the corresponding author upon reasonable request.
