# Supplementary material for: Impact of oral/dental disease burden on postoperative infective complications: a prospective cohort study
Source: Clin Oral Investig. 2023 Sep 20;27(11):6461–70. doi: 10.1007/s00784-023-05251-4 (PMC10630249; doi:10.1007/s00784-023-05251-4)
Supplement: Supplementary file 1 — Supplementary file1 (DOCX 53 KB) [file 784_2023_5251_MOESM1_ESM.docx]

Appendix Table 1 Standardised indices used for preoperative dental assessment

| Index | Description |
| --- | --- |
| (a) Decayed, Missing and Filled Teeth Index (DMFT) [20] | determines the total number of permanent teeth that have past or present carious lesions. |
| (b) Periodontal Screening and Recording Index (PSR) [21] | is used to survey and detect periodontal disease and treatment needs. Periodontal pocket depths are assessed on all teeth and the maximum pocket depth per tooth recorded. Scores (0) absence of clinical sign; (1) bleeding on probing; (2) supra and/or subgingival calculus and/or defective margins; (3) periodontal pocket 4 mm to 5.5 mm deep (coloured band on probe partially visible); (4) periodontal pocket 6mm deep (colored band no longer visible); (*) periodontal abnormalities present. |
| (c) Oral Hygiene Index (OHI) [22] | allows for classification of oral hygiene of dentate patients by quantifying the plaque and calculus index. Widely used in epidemiological studies of periodontal diseases to evaluate individual level of oral hygiene and efficacy of toothbrushing. |
| (d) Tongue Plaque Index [23] | visually evaluates tongue plaque status on the surface of the tongue and allows for classification of oral hygiene of edentulous patients. The tongue has been reported to be a reservoir of oral bacteria and edentulous patients with the presence of tongue plaque coating have demonstrated significantly higher salivary bacterial counts than those with no tongue coating. Scores (0) indicates no visible tongue coating; (1) indicates presence of visible tongue coating. |
| (e) The Challacombe Scale [24] | serves as the Clinical Oral Dryness Score – includes 10 characteristics: 1) mirror sticks to oral mucosa; 2) mirror sticks to tongue; 3) saliva frothy; 4) no saliva pooling in floor of mouth; 5) tongue shows generalized shortened papillae (mild depapillation); 6) altered gingival architecture (i.e. smooth); 7) glassy appearance of oral mucosa, especially palate; 8) tongue lobulated/fissured; 9) cervical caries (more than two teeth); 10) debris on palate or sticking to teeth. One score is assigned to each sign. Scores 1–3 indicate a mild, 4–6 a moderate and 7–10 a severe oral dryness. |
